# *Coronavirus HKU15* in respiratory tract of pigs and first discovery of coronavirus quasispecies in 5′-untranslated region

**DOI:** 10.1038/emi.2017.37

**Published:** 2017-06-21

**Authors:** Patrick CY Woo, Susanna KP Lau, Chi-Ching Tsang, Candy CY Lau, Po-Chun Wong, Franklin WN Chow, Jordan YH Fong, Kwok-Yung Yuen

**Affiliations:** 1 Department of Microbiology, The University of Hong Kong, Hong Kong, China; 2 Research Centre of Infection and Immunology, The University of Hong Kong, Hong Kong, China; 3 State Key Laboratory of Emerging Infectious Diseases, The University of Hong Kong, Hong Kong, China; 4 Carol Yu Centre for Infection, The University of Hong Kong, Hong Kong, China; 5 Collaborative Innovation Centre for Diagnosis and Treatment of Infectious Diseases, The University of Hong Kong, Hong Kong, China

**Keywords:** coronavirus, infection, pig, respiratory tract

## Abstract

*Coronavirus HKU15* is a deltacoronavirus that was discovered in fecal samples of pigs in Hong Kong in 2012. Over the past three years, *Coronavirus HKU15* has been widely detected in pigs in East/Southeast Asia and North America and has been associated with fatal outbreaks. In all such epidemiological studies, the virus was generally only detected in fecal/intestinal samples. In this molecular epidemiology study, we detected *Coronavirus HKU15* in 9.6% of the nasopharyngeal samples obtained from 249 pigs in Hong Kong. Samples that tested positive were mostly collected during winter. Complete genome sequencing of the *Coronavirus HKU15* in two nasopharyngeal samples revealed quasispecies in one of the samples. Two of the polymorphic sites involved indels, but the other two involved transition substitutions. Phylogenetic analysis showed that the two nasopharyngeal strains in the present study were most closely related to the strains PDCoV/CHJXNI2/2015 from Jiangxi, China, and CH/Sichuan/S27/2012 from Sichuan, China. The outbreak strains in the United States possessed highly similar genome sequences and were clustered monophyletically, whereas the Asian strains were more diverse and paraphyletic. The detection of *Coronavirus HKU15* in respiratory tracts of pigs implies that in addition to enteric infections, *Coronavirus HKU15* may be able to cause respiratory infections in pigs and that in addition to fecal-oral transmission, the virus could possibly spread through the respiratory route. The presence of the virus in respiratory samples provides an alternative clinical sample to confirm the diagnosis of *Coronavirus HKU15* infection. Quasispecies were unprecedentedly observed in the 5′-untranslated region of coronavirus genomes.

## INTRODUCTION

Coronaviruses (CoVs) are found in a wide variety of animals, in which they can lead to enteric, hepatic, neurological and respiratory illnesses of differing severity. On the basis of genotypic and serological characterization, CoVs were traditionally divided into three distinct groups. In 2009, the Coronavirus Study Group of the International Committee for Taxonomy of Viruses replaced the traditional CoV groups 1, 2 and 3 with three genera, *Alphacoronavirus*, *Betacoronavirus* and *Gammacoronavirus*, respectively.^1^ In the same year, we discovered three novel CoVs in avian cloacal swabs.^2^ These CoVs formed a distinct novel CoV genus, named *Deltacoronavirus.*^1^ Subsequently, in a large epidemiological study, we discovered seven additional deltacoronaviruses.^3^ Interestingly, one of these deltacoronaviruses, which was originally named porcine CoV HKU15, was found in fecal samples of pigs in Hong Kong, and it is the only mammalian deltacoronavirus.^3^ In 2016, the Coronavirus Study Group of the International Committee for Taxonomy of Viruses rectified the species name for this virus to *Coronavirus HKU15.*^4^

Over the past three years, *Coronavirus HKU15* has been widely detected in pigs in East/Southeast Asia and North America and was found to be associated with fatal outbreaks.^5, 6, 7, 8, 9, 10, 11, 12, 13, 14, 15, 16, 17, 18, 19, 20, 21, 22, 23^ In these epidemiological studies, *Coronavirus HKU15* was generally only detected in fecal/intestinal samples. However, some CoVs, such as transmissible gastroenteritis CoV (TGEV)/porcine respiratory CoV (PRCV) of *Alphacoronavirus 1* and bovine CoV of *Betacoronavirus 1*, could be detected consistently in both fecal and respiratory samples.^24, 25^ Therefore, we hypothesized that *Coronavirus HKU15* may also be present in respiratory samples of pigs, which has implications for its transmission and potential role in respiratory diseases and for the use of nasopharyngeal sampling as an alternative method of identifying infected pigs. To test this hypothesis, we performed a molecular epidemiology study on nasopharyngeal samples collected from pigs in Hong Kong. Two complete genomes of the ‘respiratory’ *Coronavirus HKU15* were sequenced, and comparative genomic and phylogenetic studies were performed. The implications of the presence of *Coronavirus HKU15* in respiratory samples are also discussed.

## MATERIALS AND METHODS

### Sample collection

Nasopharyngeal samples from pigs were collected in Hong Kong over a 26-month period (January 2012–February 2014). These samples were obtained from slaughterhouses and pig farms in Hong Kong with the assistance of the Veterinary Public Health Section, Food and Environmental Hygiene Department; and the Agriculture, Fisheries and Conservation Department of the Government of Hong Kong. Immediately after sample collection, each nasopharyngeal swab was submerged in viral transport medium for viral transport and maintenance.

### RNA extraction and reverse transcription

Viral RNAs were extracted from the nasopharyngeal samples of pigs by using 200 μL of inoculated viral transport medium for each sample and by utilizing the EZ1 Advanced XL system (Qiagen, Hilden, Germany) and EZ1 Virus Mini Kit v2.0 (Qiagen) according to the manufacturer’s protocol with RNase-free water as the eluent. Reverse transcription (RT) was performed using the SuperScript III Reverse Transcriptase (Invitrogen, Carlsbad, CA, USA) according to the manufacturer’s protocol by random priming.

### *Coronavirus* HKU15 screening

Detection of *Coronavirus HKU15* was performed by polymerase chain reaction (PCR) amplifying a 289-bp fragment of the RNA-dependent RNA polymerase (RdRp) gene, using the specific primer pair LPW14077 (5′-ACA CAC TTG CTG TAA CCA AA-3′) and LPW14080 (5′-ATC ATT AGA GTC ACC ACG AT-3′). PCR and DNA sequencing were carried out following our previous publications with slight modification.^26, 27^ Briefly, each PCR mixture contained PCR buffer (50 mM of KCl, 10 mM of Tris-HCl at pH 8.3 and 3 mM of MgCl_2_; Applied Biosystems, Foster City, CA, USA), 200 μM of each deoxynucleoside triphosphate (Roche Diagnostics, Basel, Switzerland), 1 μM of each primer (Invitrogen), 1.0 U of AmpliTag Gold DNA polymerase (Applied Biosystems) and cDNA. The mixtures were subjected to 60 thermocycles of 94 °C for 1 min, 50 °C for 1 min and 72 °C for 1 min, with an initial denaturation at 95 °C for 10 min and a final extension at 72 °C for 10 min for DNA amplification using the GeneAmp PCR System 9700 automated thermal cycler (Applied Biosystems). Standard precautions were taken to avoid contamination, and no false-positive result was observed for the negative controls. PCR products were agarose gel-purified using the QIAquick Gel Extraction kit (Qiagen). Both strands of the PCR products were sequenced twice by the ABI Prism 3130*xl* Genetic Analyzer (Applied Biosystems) using the two PCR primers.

### Complete genome sequencing

The genomes of two *Coronavirus HKU15* strains detected in the nasopharyngeal samples of two different pigs were sequenced following our previous publications^26, 27^ with modifications. Briefly, viral RNAs were converted to cDNAs using SuperScript III reverse transcriptase with a combined random priming and oligo(dT) priming strategy. The cDNAs were PCR-amplified by primers (Supplementary Tables S1 and S2) that were designed by multiple alignment of the genome sequences of other *Coronavirus HKU15* strains with complete genomes available or from the results of the first and subsequent rounds of PCR–sequencing. PCRs were performed using the iProof High-Fidelity PCR kit (Bio-Rad Laboratories, Hercules, CA, USA) according to the manufacturer’s protocol, and DNA sequencing was performed as mentioned above. When ambiguous peaks were observed consistently in the electropherograms after several attempts of PCR-sequencing for certain genomic regions, cloning followed by plasmid sequencing was performed according to our previous publication,^28^ except the Zero Blunt TOPO PCR Cloning kit (Invitrogen) was used to resolve the sequence ambiguities. PCRs using recombinant plasmids as templates were also performed to confirm that indels at mononucleotide polymeric regions were not the result of polymerase slippage. The 5′ ends of the viral genomes were amplified and sequenced by the rapid amplification of cDNA ends (RACE) using the SMARTer RACE 5′/3′ kit (Clontech Laboratories, Mountain View, CA, USA) according to the manufacturer’s protocol with the reverse primers LPW15379 (5′-TGG GTA ATG TGT CCG CTG ACG GGC GGT G-3′), LPW15380 (5′-AGA AGT GGT GGA TGG TCA GAG GAA CGG T-3′) and LPW16265 (5′-GTG GCT GGT TTC CAG GTA ATC TA-3′). The sequences of the PCR products were then assembled manually to obtain the genomes of the two nasopharyngeal strains.

### Phylogenetic characterization

Pairwise alignment was performed using BioEdit 7.2.5 (optimal GLOBAL alignment)^29^ or EMBOSS Stretcher (Nucleotide Alignment);^30^ whereas multiple sequence alignment was performed using MUSCLE 3.8,^31^ where the aligned sequences were further manually inspected and edited. Tests for substitution models and phylogenetic analysis by the maximum likelihood method were performed using MEGA 6.0.6.^32^

### Estimation of divergence times

Divergence times for the *Coronavirus HKU15* strains were calculated based on the complete genome sequence data, utilizing the Bayesian Markov chain Monte Carlo method using BEAST 1.8.0^33^ with the substitution model GTR (general time-reversible model)+G (gamma-distributed rate variation)+I (estimated proportion of invariable sites), a strict molecular clock, and a constant coalescent. Fifty million generations were run with trees sampled every 1000th generation to yield 50 000 trees. Convergence was assessed based on the effective sampling size after a 10% burn-in using Tracer 1.6.0. The mean time to the most recent common ancestor (tMRCA) and the highest posterior density (HPD) regions at 95% were calculated. The trees, after a 10% burn-in, were summarized as a single tree using TreeAnnotator 1.8.0 by choosing the tree with the maximum sum of posterior probabilities (maximum clade credibility) and viewed using FigTree 1.4.0.

### Nucleotide sequence accession number

The complete genome sequences of the two nasopharyngeal *Coronavirus HKU15* strains were deposited into the International Nucleotide Sequence Databases with accession numbers LC216914 and LC216915.

## RESULTS

### Animal surveillance

**Figure 1:**
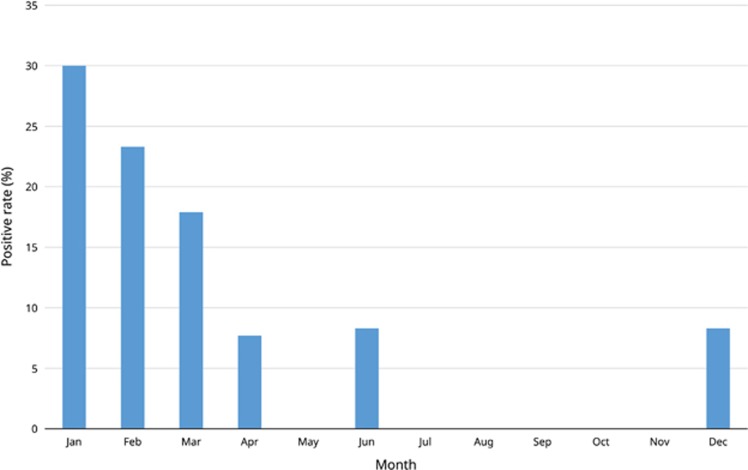
Seasonal variation in the detection rate of *Coronavirus HKU15* in swine nasopharynx during January 2012–February 2014.

A total of 249 nasopharyngeal samples from 249 pigs were tested. RT-PCR for a 289-bp fragment of the RdRp gene of *Coronavirus HKU15* was positive in 24 (9.6%) of the 249 nasopharyngeal samples. The samples that tested positive were mostly collected during winter (December–March) (Figure 1). DNA sequencing showed that seven sequence variants were detected among the 24 positive samples, and pairwise alignment showed that these seven sequence variants possessed 98.7%–100% sequence identity to the corresponding region in the RdRp gene of *Coronavirus HKU15* strain HKU15-155 that we previously found in fecal samples of pigs in Hong Kong^3^ (Supplementary Figure S1).

### Complete genome sequencing and genome analysis

**Figure 2:**
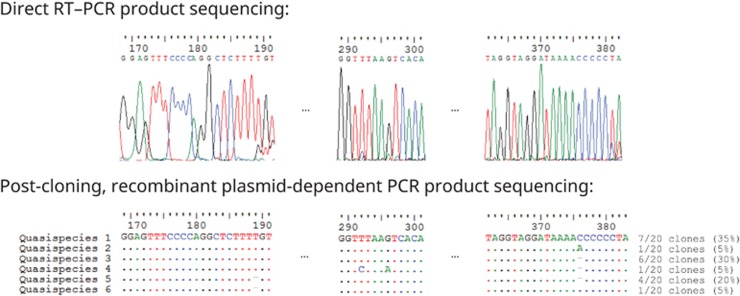
Resolution of sequence ambiguities by cloning. RT, reverse transcription; PCR, polymerase chain reaction. Numbers above nucleotides indicate the respective nucleotide positions with respect to the complete genome sequence of *Coronavirus HKU15* strain S579N quasispecies 1 (International Nucleotide Sequence Databases LC216914). Six intra-strain quasispecies were found. Post-cloning plasmid-dependent PCR-sequencing confirmed that the presence of indels at positions 189 and 376 was not due to polymerase slippage. Quasispecies 1 and 3 were detected in both nested PCR using first round primers LPW18323/LPW30836 and second round primers LPW33264/LPW6975 as well as nested PCR using first round primers LPW33199/LPW33200 and second round primers LPW33264/LPW6975. However, quasispecies 2 and 4 were only detected in nested PCR using first round primers LPW33199/LPW33200 and second round primers LPW33264/LPW6975, whereas quasispecies 5 and 6 were only detected in nested PCR using first round primers LPW18323/LPW30836 and second round primers LPW33264/LPW6975.

Complete genome sequencing was performed for the *Coronavirus HKU15* found in two of the positive nasopharyngeal samples (S579N and S582N). Excluding the 3′ poly(A) tail, the genomes of S579N and S582N were 25 411–25 413 and 25 397 nucleotides long, respectively. The genome organization of the two strains was the same as that of other *Coronavirus HKU15* strains. The lengths of the seven open reading frames (ORFs) of the two strains S579N/S582N were 18 803/18 788, 3480, 252, 654, 285, 1029 and 603 bp, respectively. The genomes of the two strains possessed 98.9%–99.2% sequence identity to that of the representative isolate HKU15-155. Quasispecies were detected in one of the samples (S579N) at the 5′ genomic region via two independent nested PCRs targeting the 122nd–505th bases of the genome using two different primer pairs for the first round and the same primer pair for the second round of reaction. For S579N, direct sequencing of the PCR products yielded ambiguous peaks in the sequencing electropherograms, which could only be resolved after cloning (Figure 2). Post-cloning DNA sequencing revealed that there were six sequence variants, with four polymorphic sites, for this genomic region (Figure 2). Two of the polymorphic sites, located at the 189th and 376th nucleotide positions, involved indels (ΔT and ΔA/C, respectively), whereas the other two polymorphic sites, located at the 292nd and 296th nucleotide positions, involved transition substitutions (T→C and G→A, respectively). Additionally, PCR–DNA sequencing using the recombinant plasmids as amplification templates did not generate the same sequence ambiguities observed in the pre-cloning experiment.

### Phylogenetic characterization

**Figure 3:**
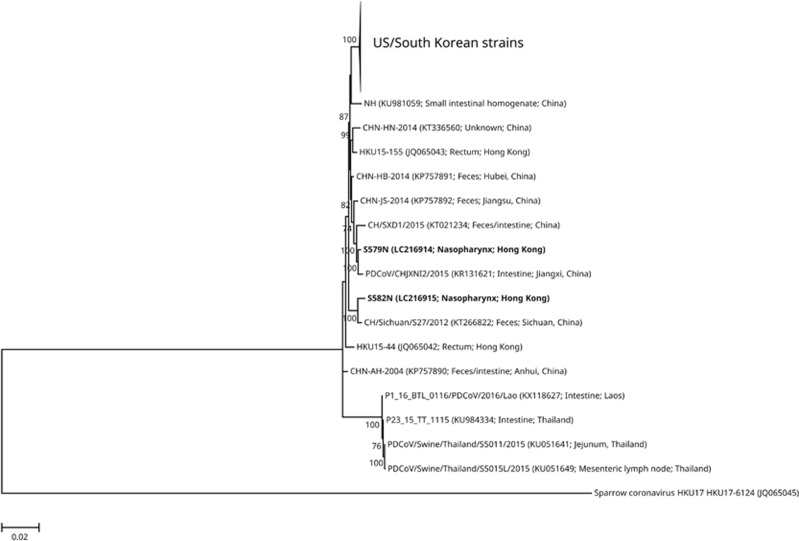
Phylogenetic tree showing the relationship of the two *Coronavirus HKU15* nasopharyngeal strains to other *Coronavirus HKU15* strains. The trees were inferred from the almost complete genome sequence data by the maximum likelihood method with the substitution model TN93 (Tamura–Nei model)+G (gamma-distributed rate variation)+I (estimated proportion of invariable sites). The scale bar indicates the estimated number of substitutions per base. Numbers at nodes (expressed in percentage) indicate the levels of bootstrap support calculated from 1 000 replicates, and values lower than 70 are not shown. The two nasopharyngeal strains sequenced in this study are highlighted in bold. All names and accession numbers are given as cited in the International Nucleotide Sequence Databases.

Phylogenetic analysis of the complete genomes of the two nasopharyngeal strains and other *Coronavirus HKU15* strains showed that the outbreak strains in the United States possessed highly similar genome sequences and that they were all clustered together monophyletically, whereas the Asian strains were more diverse and paraphyletic, with the Lao and Thai strains occupying the basal lineage; however, the South Korean strain KNU14-04 was more similar to the US strains than to the other Asian strains (Figure 3 and Supplementary Figure S2). The two nasopharyngeal strains S579N and S582N in the present study were most closely related to strains PDCoV/CHJXNI2/2015 from Jiangxi, China and CH/Sichuan/S27/2012 from Sichuan, China (Figure 3 and Supplementary Figure S2). Similar to other Chinese and Hong Kong strains (except CHN-AH-2004 and HKU15-44) and a Thai strain (P23_15_TT_1115), there was a deletion of an asparagine residue of the asparagine trimer located at the 50th–52nd amino-acid positions in the S protein compared with the other strains from South Korea, Laos and United States and to two other strains from Thailand. The deletion of this codon has been proposed as a genetic marker for the differentiation of China and Hong Kong strains from US and South Korean strains.^12^

### Estimation of divergence dates

**Figure 4:**
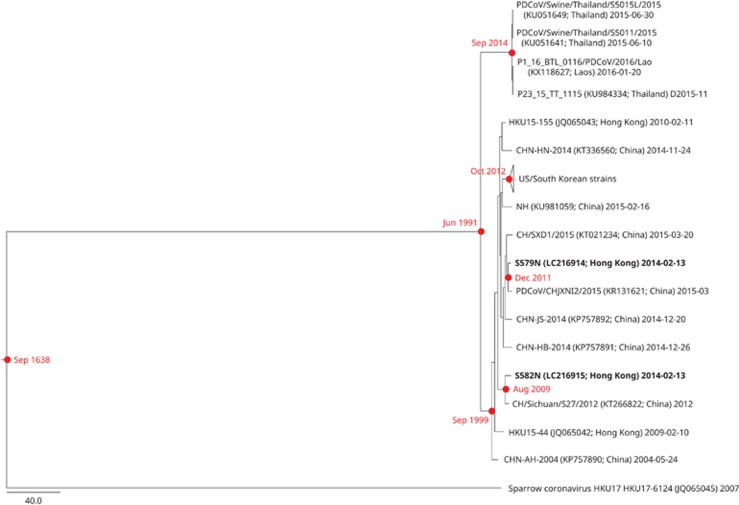
Estimation of tMRCA for *Coronavirus HKU15*. The timescaled phylogeny was inferred from complete *Coronavirus HKU15* genomes. The two nasopharyngeal strains sequenced in this study are highlighted in bold. All names and accession numbers are given as cited in the International Nucleotide Sequence Databases.

The estimated mean evolutionary rate of the complete genome sequence data set was 6.549 × 10^−4^ (95% HPD: 5.632–7.476 × 10^−4^) substitutions per site per year, which is approximately 1.7-fold higher than that estimated in a previous study.^19^ The root of the tree was September 1638 (95% HPD: June 1570–March 1698). The tMRCA of the diversity of *Coronavirus HKU15* was dated to June 1991 (95% HPD: November 1987–June 1994); and the tMRCA of the Thai/Laos strains was traced back to September 2014 (95% HPD: May 2014–January 2015). The tMRCAs for the clade containing US/Korean strains was estimated to be October 2012 (95% HPD: June 2012–January 2013), which is slightly delayed compared with that estimated in a previous study.^19^ For the two nasopharyngeal strains characterized in this study (S579N and S582N), they were estimated to have diverged from their respective MRCAs in December 2011 (95% HPD: May 2011–July 2012) and August 2009 (95% HPD: November 2008–April 2010), respectively (Figure 4).

## DISCUSSION

*Coronavirus HKU15* was detected in nasopharyngeal samples of pigs. Although *Coronavirus HKU15* has been widely detected in various locations around the Pacific Ocean, including Canada,^7^ China,^12, 13, 16, 17^ Hong Kong,^3^ Laos,^22, 23^ Mexico,^19^ South Korea,^5, 21^ Thailand,^20, 23^ Vietnam^23^ and the United States,^6, 7, 8, 9, 10, 11, 14, 15, 18, 19^ the virus has principally been found in fecal or intestinal specimens. There have been a few exceptional circumstances; in one study, the presence of *Coronavirus HKU15* was reported in the blood, liver, lung and kidney of one pig,^15^ and in a few other studies, *Coronavirus HKU15* was found to exist in the blood (*n*=10), mesenteric lymph node (*n*=2) and saliva (*n*=10)/oral fluid (*n*=73) of pigs,^7, 19, 20^ implying that *Coronavirus HKU15* can cause systemic infections in occasional cases. In this study, *Coronavirus HKU15* was found in 9.6% of the nasopharyngeal samples of pigs, which is similar to the 10.1% positive rate of *Coronavirus HKU15* in fecal samples of pigs that we reported previously.^3^ Seasonal variation in the detection rate of *Coronavirus HKU15* from pigs was noted, where most of the positive samples were collected in winter. This is similar to the pattern of seasonal variation in a surveillance study carried out in the United States, where the detection rate for *Coronavirus HKU15* was much lower during summer.^19^ It has recently been confirmed that *Coronavirus HKU15* is able to cause swine enteric infections by infecting gnotobiotic and conventional pigs with *Coronavirus HKU15*.^15^ The detection of *Coronavirus HKU15* in respiratory tracts of pigs has the following implications. First, in addition to enteric infections, *Coronavirus HKU15* may be able to cause respiratory infections in pigs. Second, in addition to fecal-oral transmission, the virus may be able to spread through the respiratory route. Third, the presence of the virus in respiratory samples provides an alternative clinical sample to confirm the diagnosis of *Coronavirus HKU15* infection. Further studies will determine the full spectrum of clinical diseases and pathologies associated with *Coronavirus HKU15.*

From the data of the present study, both the ‘enteric’ and ‘respiratory’ *Coronavirus HKU15* may possess similar properties. A number of animal CoVs possess dual or multiple tissue tropisms. For example, TGEV, which is another enteropathogenic CoV that infects pigs, could also be found in the nasopharynx of pigs as PRCV, which is a deletion mutant of TGEV.^25^ Moreover, bovine CoV is both an enteric and a respiratory pathogen in cattle.^24^ Similar to TGEV/PRCV, *Coronavirus HKU15* is recovered from both respiratory and gastrointestinal samples. However, unlike TGEV/PRCV, in which there is a 621–681 nucleotide deletion at the 5′ end of the spike (S) gene leading to a loss of 1–2 antigenic sites in PRCV,^34^ comparative genome analysis of *Coronavirus HKU15* from respiratory and fecal samples did not show any obvious difference in their S proteins or other parts of their genomes. Phylogenetic analysis also did not reveal a separate clustering of fecal/intestinal and nasopharyngeal isolates (Figure 2). Further cell culture experiments are required to confirm whether all strains of this species possess intrinsic tropism to both enteric and respiratory tissues.

This is also the first report of CoV quasispecies in the 5′-untranslated region (UTR). In one (S579N) of the two *Coronavirus HKU15* genomes that we sequenced in this study, variant sites were observed at four positions; two of them were due to nucleotide substitutions, and the other two were results of indels at mononucleotide polymeric regions (189th and 376th bases). These two indels were genuine variant sites instead of being due to polymerase slippage during the amplification process because recombinant plasmid-dependent PCR–sequencing no longer resulted in sequence ambiguities in the electropherograms. Although the existence of quasispecies has been reported in CoVs, the variant sites were found in coding regions or 3′-UTR.^35, 36, 37, 38^ In the case of severe acute respiratory syndrome-related coronavirus, all of the variant sites observed in the quasispecies were located at the S gene.^35^ For bovine CoV, one of the two strains with naturally occurring intra-isolate quasispecies had all seven variant sites located at ORF1a, whereas for the other strain with naturally occurring intra-isolate quasispecies, there were 85 polymorphic sites scattered across ORF1a (*n*=28), ORF1b (*n*=19), 32 kDa-non-structural protein (NSP) gene (*n*=6), hemagglutinin esterase (HE) gene (*n*=2), S gene (*n*=18), 4.9 kDa-NSP gene (*n*=2), 4.8 kDa-NSP gene (*n*=2), membrane (M) gene (*n*=2), nucleocapsid (N) gene (*n*=5) and 3′-UTR (*n*=1).^36^ Similar to bovine CoV, Middle East respiratory syndrome-related coronavirus also possessed all the intra-host single nucleotide variations throughout its genome except the 5′-UTR.^37, 38^ In this study, all four variant sites (189 ΔT, 292 T→C, 296 G→A and 376 ΔA/C) were present in the 5′-UTR and were not located in the leader sequence or the transcription regulatory sequence. We speculate that the existence of quasispecies in CoVs may play a role in CoV evolution, in addition to the more well-known high-recombination and mutation rates in CoV genomes.^39^
