# Supplementary material for: Coronavirus HKU15 in respiratory tract of pigs and first discovery of coronavirus quasispecies in 5′-untranslated region
Source: Emerg Microbes Infect. 2017 Jun 21;6(6):e53–. doi: 10.1038/emi.2017.37 (PMC5584481; doi:10.1038/emi.2017.37)
Supplement: Supplementary Figure S1 [file emi201737x1.pdf]

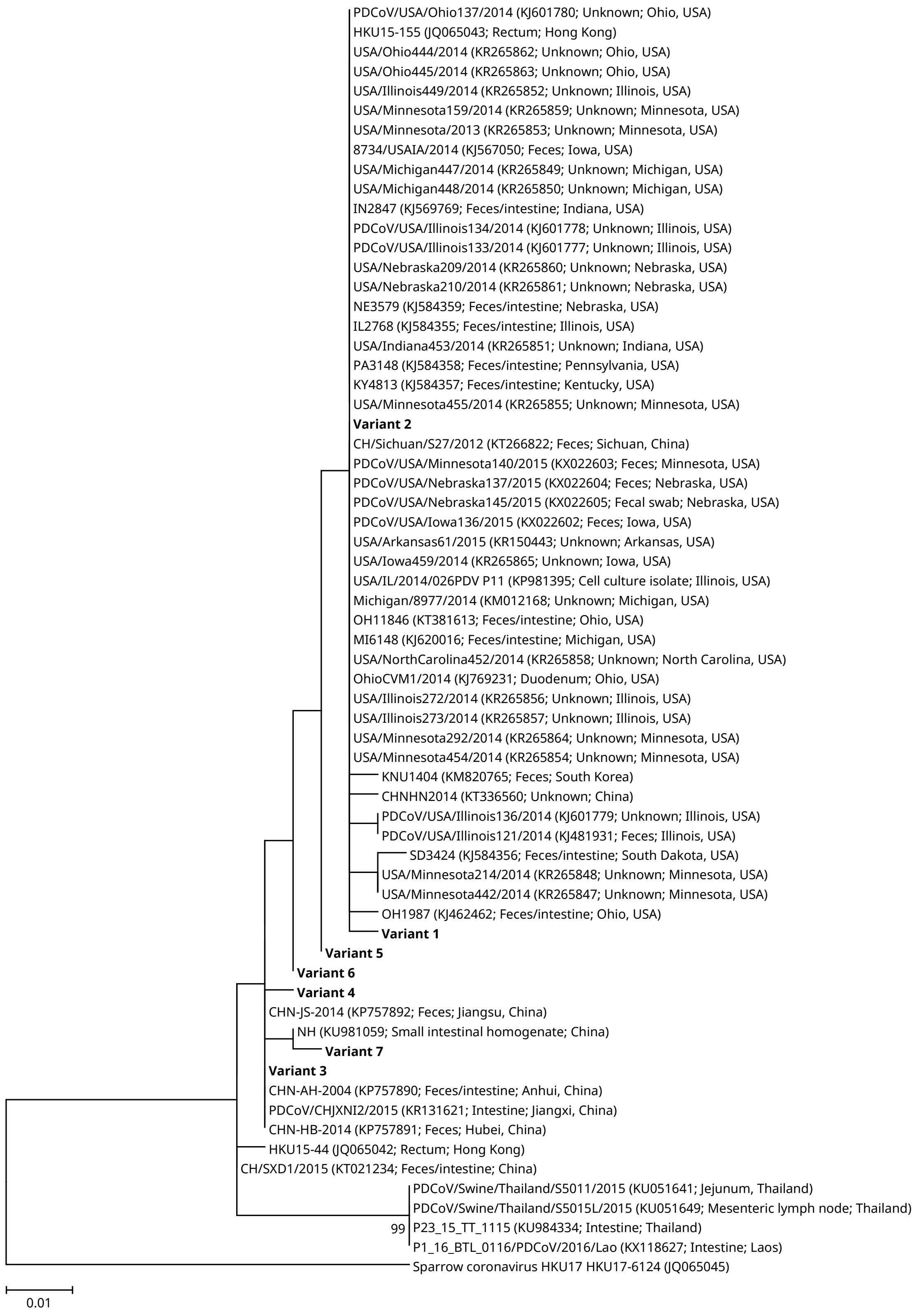

Supplementary Figure S1

Supplementary Figure S1 Phylogenetic tree showing the relationship of *Coronavirus HKU15* strains detected from swine nasopharynx in Hong Kong to other *Coronavirus HKU15* strains. The tree was inferred from the partial RNA-dependent RNA polymerase (RdRp) gene sequence data by the maximum likelihood method with the substitution model K2 (Kimura 2–parameter model). The scale bar indicates the estimated number of substitutions per base. Numbers at nodes (expressed in percentage) indicate levels of bootstrap support calculated from 1,000 replicates, and values lower than 70 are not shown. The seven sequence variants of nasopharyngeal origins included in this study are highlighted in bold. All names and accession numbers are given as cited in the International Nucleotide Sequence Databases.
