# Supplementary material for: Coronavirus HKU15 in respiratory tract of pigs and first discovery of coronavirus quasispecies in 5′-untranslated region
Source: Emerg Microbes Infect. 2017 Jun 21;6(6):e53–. doi: 10.1038/emi.2017.37 (PMC5584481; doi:10.1038/emi.2017.37)
Supplement: Supplementary Figure S2 [file emi201737x2.pdf]

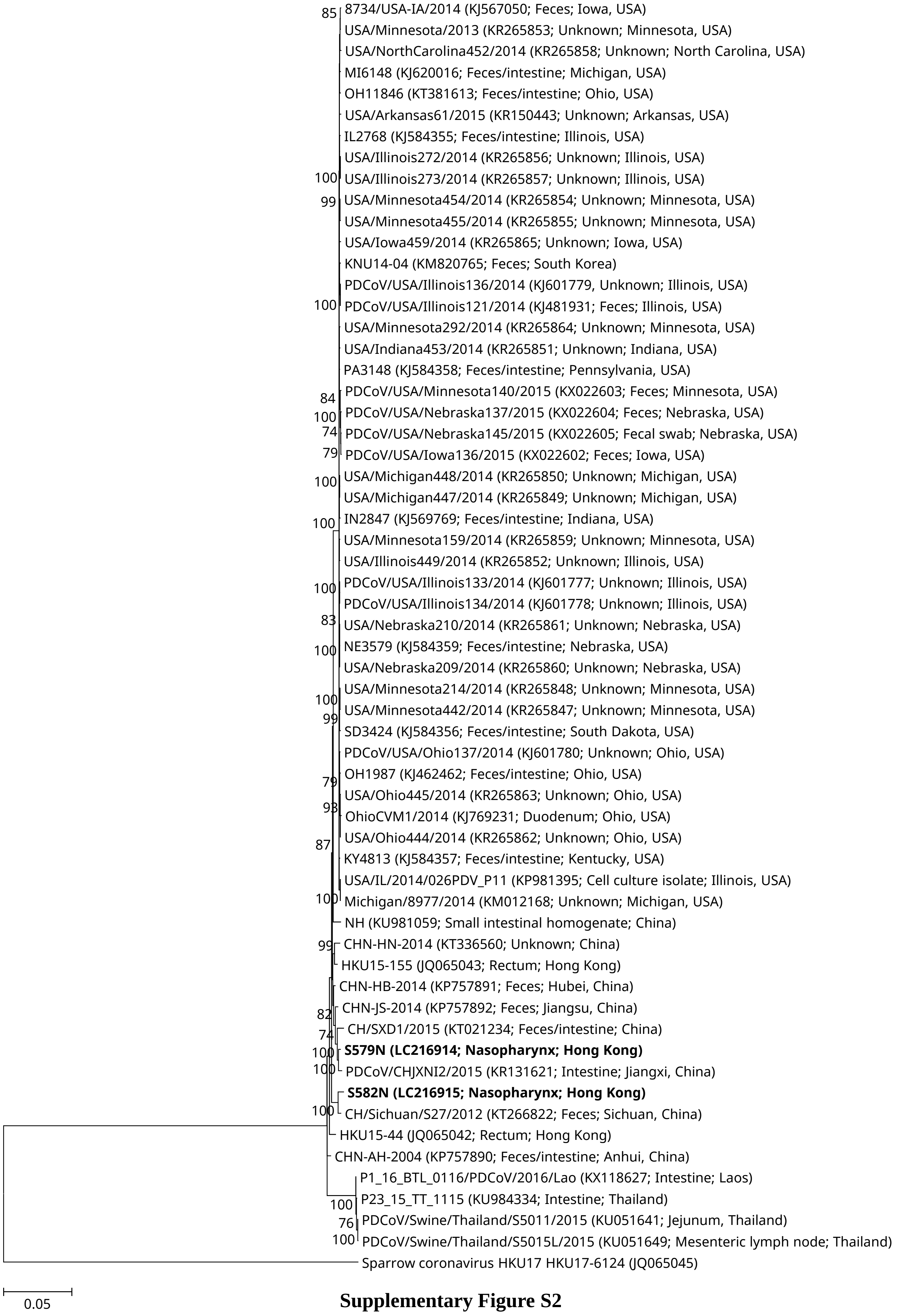

Supplementary Figure S2

Supplementary Figure S2 Phylogenetic tree showing the relationship of the two *Coronavirus HKU15* nasopharyngeal strains to other *Coronavirus HKU15* strains. The trees were inferred from the almost complete genome sequence data by the maximum likelihood method with the substitution model TN93 (Tamura–Nei model) + G (gamma-distributed rate variation) + I (estimated proportion of invariable sites). The scale bar indicates the estimated number of substitutions per base. Numbers at nodes (expressed in percentage) indicate levels of bootstrap support calculated from 1,000 replicates, and values lower than 70 are not shown. The two nasopharyngeal strains sequenced in this study are highlighted in bold. All names and accession numbers are given as cited in the International Nucleotide Sequence Databases.
