# Supplementary material for: Coronavirus HKU15 in respiratory tract of pigs and first discovery of coronavirus quasispecies in 5′-untranslated region
Source: Emerg Microbes Infect. 2017 Jun 21;6(6):e53–. doi: 10.1038/emi.2017.37 (PMC5584481; doi:10.1038/emi.2017.37)
Supplement: Supplementary Table S1 [file emi201737x3.pdf]

**Supplementary Table S1** PCR primers used for complete genome sequencing

| Region | Primers for 1st round of PCR | Primer position with respect to the genome of *Coronavirus HKU15* HKU15–155 (JQ065043) | Primers for 2nd round of PCR | Primer position with respect to the genome of *Coronavirus HKU15* HKU15–155 (JQ065043) |
|---|---|---|---|---|
| 1–S579N | LPW18323 | 65–83 | LPW33264 | 122–142 |
| | LPW30836 | 580–601 | LPW6975 | 483–505 |
| | LPW33199 | 50–70 | LPW33264 | 122–142 |
| | LPW33200 | 602–622 | LPW6975 | 483–505 |
| 1–S582N | LPW18323 | 65–83 | | |
| | LPW30836 | 580–601 | | |
| 2 | LPW18326 | 484–506 | | |
| | LPW13737 | 1614–1641 | | |
| 3 | LPW29668 | 1398–1419 | LPW29668 | 1398–1419 |
| | LPW29669 | 3072–3092 | LPW15250 | 2742–2769 |
| 4 | LPW30818 | 2593–2614 | | |
| | LPW30758 | 2908–2929 | | |
| 5 | LPW26394 | 2736–2754 | | |
| | LPW26393 | 4177–4196 | | |
| 6 | LPW30876 | 3543–3564 | | |
| | LPW30877 | 3849–3869 | | |
| 7 | LPW29670 | 3389–3410 | | |
| | LPW29671 | 4916–4937 | | |
| 8 | LPW30733 | 4500–4521 | | |
| | LPW30814 | 4948–4969 | | |
| 9 | LPW29672 | 4840–4860 | | |
| | LPW29673 | 6380–6401 | | |
| 10 | LPW30735 | 6210–6227 | | |
| | LPW30736 | 6917–6938 | | |
| 11 | LPW29674 | 6289–6310 | LPW29674 | 6289–6310 |
| | LPW29675 | 7707–7726 | LPW10595 | 7551–7573 |
| 12 | LPW30878 | 7247–7265 | | |
| | LPW30879 | 7846–7865 | | |
| 13 | LPW29676 | 7567–7588 | LPW29676 | 7567–7588 |
| | LPW29677 | 8267–8289 | LPW10502 | 8247–8269 |
| 14 | LPW14878 | 7858–7879 | | |
| | LPW10062 | 9026–9048 | | |
| 15 | LPW30737 | 8881–8902 | | |
| | LPW30815 | 9711–9728 | | |
| 16 | LPW29678 | 8909–8929 | | |
| | LPW29679 | 10385–10407 | | |
| 17 | LPW29680 | 10310–10330 | LPW29680 | 10310–10330 |
| | LPW29681 | 11784–11805 | LPW7430 | 11163–11185 |
| 18–S579N | LPW30924 | 11026–11048 | | |
| | LPW30925 | 11852–11870 | | |
| 18–S582N | LPW30739 | 11007–11027 | | |
| | LPW30740 | 11886–11907 | | |
| 19 | LPW29682 | 11651–11673 | | |
| | LPW29683 | 13122–13141 | | |
| 20 | LPW30741 | 12936–12954 | | |
| | LPW30742 | 13253–13274 | | |
| 21 | LPW29384 | 13072–13093 | | |
| | LPW29685 | 14072–14093 | | |
| 22 | LPW9763 | 13528–13550 | | |
| | LPW26389 | 14863–14879 | | |
| 23 | LPW29686 | 14629–14648 | | |
| | LPW29687 | 16097–16116 | | |
| 24 | LPW30743 | 15893–15912 | | |
| | LPW30744 | 17648–17670 | | |
| 25 | LPW11164 | 17455–17477 | | |
| | LPW14273 | 18977–18996 | | |
| 26 | LPW30745 | 17978–17996 | | |
| | LPW30746 | 18448–18467 | | |
| 27 | LPW26388 | 18293–18311 | | |
| | LPW26387 | 19807–19826 | | |

## Supplementary Table S1 (continued)

| Region | Primers for 1st round of PCR | Primer position with respect to the genome of *Coronavirus HKU15* HKU15–155 (JQ065043) | Primers for 2nd round of PCR | Primer position with respect to the genome of *Coronavirus HKU15* HKU15–155 (JQ065043) |
|---|---|---|---|---|
| 28 | LPW29896 | 18876–18899 | LPW29896 | 18876–18899 |
| | LPW29897 | 20418–20439 | LPW14271 | 20394–20415 |
| 29 | LPW14444 | 19604–19624 | | |
| | LPW14272 | 20301–20320 | | |
| | LPW26386 | 21193–21210 | | |
| 30 | LPW14445 | 21047–21069 | | |
| | LPW7184 | 22007–22029 | | |
| 31 | LPW29900 | 21015–21036 | | |
| | LPW10932 | 21459–21482 | | |
| | LPW29901 | 22500–22521 | | |
| 32 | LPW29902 | 22352–22374 | | |
| | LPW29903 | 23847–23870 | | |
| 33 | LPW29904 | 23627–23647 | LPW29904 | 23627–23647 |
| | LPW29905 | 24773–24796 | LPW6038 | 24145–24167 |
| | | | LPW14448 | 23934–23953 |
| | | | LPW29905 | 24773–24796 |
| 34 | LPW26396 | 24580–24599 | | |
| | LPW418 | 3′ polyA | | |
