# Supplementary material for: Coronavirus HKU15 in respiratory tract of pigs and first discovery of coronavirus quasispecies in 5′-untranslated region
Source: Emerg Microbes Infect. 2017 Jun 21;6(6):e53–. doi: 10.1038/emi.2017.37 (PMC5584481; doi:10.1038/emi.2017.37)
Supplement: Supplementary Table S2 [file emi201737x4.pdf]

Supplementary Table S2 Sequences of the PCR primers

Primer	Sequence
LPW6038	5′–CCACCTGGCTTTTGTCTGGTGTA–3′
LPW6975	5′–CAGAATAGGATTTCGCGCTCCTA–3′
LPW7184	5′–ACTGTGAYTTDACACACTCATTA–3′
LPW7430	5′–CTGATGCTCATTAGGTTGAGGTT–3′
LPW9763	5′–CTTAATAAGGACATTGCGGACTT–3′
LPW10062	5′–GCAAGTAGCATGGTAATAGCCAT–3′
LPW10502	5′–ACCCTGTGTTGGACACTTGACAA–3′
LPW10595	5′–CTTGTACATAAGTGCAATGCCTA–3′
LPW10932	5′–GGCATCATGGTGCTTCCTGGTGTT–3′
LPW11164	5′–GTTTACATACCACGCAATGTCAA–3′
LPW13737	5′–TATTCTTCATCAGTAGCCACGACGACAT–3′
LPW14271	5′–CTGCTGACAGGTCAAAACTACA–3′
LPW14272	5′–GGTAACACATCCTATTGTGT–3′
LPW14273	5′–TTCGGGTGAGAATGAATGTT–3′
LPW14444	5′–AACTAACCACACTCTCAGCAT–3′
LPW14445	5′–TCTTTCTGTTCAGACGGAGTATT–3′
LPW14448	5′–CATCGCTCCAAGTCATTCTT–3′
LPW14878	5′–TGGTGCTGAATTTGACCAGTAT–3′
LPW15250	5′–TCGTCGCTGCTGGTCTTCCGTTCCTGGA–3′
LPW18323	5′–CCGACACCAATCCAGGTGC–3′
LPW18326	5′–AGGAGTGTGGAATCCTATTCTGC–3′
LPW26386	5′–CCAACTGTGCAGAGGAAT–3′
LPW26387	5′–GGTAGAGTTGTGTAATGCAG–3′
LPW26388	5′–TTGTTACCTCACCTAACGC–3′
LPW26389	5′–AGCGGTGTRACAACATG–3′
LPW26393	5′–AGAGAGCATCAATAGCAGGT–3′
LPW26394	5′–TCAAGATCCAGGAACGGAA–3′
LPW26396	5′–GGTAAAACCATTTCTCAGGT–3′
LPW29668	5′–GTGGTGTTATTGGTATTGCACT–3′
LPW29669	5′–TTAAATCCGCAGGTGTTACTG–3′
LPW29670	5′–GAGTTAGTTGTTGGTGAATTGG–3′
LPW29671	5′–GGTAATTGTAGAAACGGGTCAA–3′
LPW29672	5′–CCTCGTAAAGTCGTGGAGTGT–3′
LPW29673	5′–CCTGCTGACTAAGACGTGTAAC–3′
LPW29674	5′–TGCAGAAGACCAGTCTACCATT–3′
LPW29675	5′–GCTACACACAATGAGCGCAA–3′
LPW29676	5′–GTACAAGTGCCTTGTCTTCGCA–3′
LPW29677	5′–GATTGGACATTTAGCTGAATACC–3′
LPW29678	5′–GGATCTTATGATTTCGAGTGC–3′
LPW29679	5′–TTTACCCTATCTGAGTCAAGACG–3′
LPW29680	5′–CTTGCAGAACGTGCTGAGGAT–3′
LPW29681	5′–ATGACGTAGAGCATAAGCCAAG–3′
LPW29682	5′–GCTACAACGCTCTTAGTACAGAG–3′
LPW29683	5′–TCTGATTGCGTGCAAGTGAA–3′
LPW29684	5′–ATTAGCACCATGACTAACAGGC–3′
LPW29685	5′–GATTGGAAAGCATCAAGGATAC–3′
LPW29686	5′–GCAACCGTCAAGGATGTGTA–3′
LPW29687	5′–GCCATCGGCAACTCCTACTA–3′
LPW29896	5′–TGACCTCTACTGTTGTGATGACAT–3′
LPW29897	5′–GAGAGAATGACATGCCATTGTT–3′
LPW29900	5′–GATGGCGAAGTTGAAATACCAT–3′
LPW29901	5′–GATCTGCAATCTGTGAGAGGTT–3′
LPW29902	5′–TCCTGACGTTATAGATGTCAATC–3′
LPW29903	5′–TAAGAAGTTTCCGAGGTAGTATGC–3′
LPW29904	5′–TGGACTTCACACCATAACCAC–3′
LPW29905	5′–CCTTGACTGTGATTGAGTAAGAGA–3′
LPW30733	5′–ATGAACCTACAGGTCCTGCCAT–3′
LPW30735	5′–TCCAGCGCGGTCTTGATT–3′
LPW30736	5′–GTGACAACGCATCCCTAACCAT–3′
LPW30737	5′–CACCAATAGAGATGGTGCTTGT–3′
LPW30739	5′–TTGAGTACCAGCAAAATGCCT–3′
LPW30740	5′–TGGGTTCTCAATTGGATCAAAC–3′

Supplementary Table S2 (continued)

Primer	Sequence
LPW30741	5′–CGACATGACTCATGCCGAG–3′
LPW30742	5′–AGCATGTTTGGCATAGAACGAT–3′
LPW30743	5′–TTGTGGTGTTTAGGCAGGCA–3′
LPW30744	5′–TGAGACAGGTAAGTTAGTGCGAT–3′
LPW30745	5′–TCGCCATTGACGGTGAAGA–3′
LPW30746	5′–TGCTCATCATGCCAAAGCAT–3′
LPW30758	5′–TGCTGTTTTAGTTGGTTGTTCA–3′
LPW30814	5′–TATTGTTGAGTCAAGAGCACCA–3′
LPW30815	5′–TACGCGGTGGGGACATCT–3′
LPW30818	5′–TATTCAAGCTATGGAGGGTGAG–3′
LPW30836	5′–AAGTTGCAGAGGTGGTGGTACA–3′
LPW30876	5′–CTATCTCAGGACCGCTTACCAC–3′
LPW30877	5′–CAGTAGTGGTGCGTACAATGG–3′
LPW30878	5′–TATTGCCGTATGGGGCATT–3′
LPW30879	5′–CAGCACCAGCGAGGTCTTTA–3′
LPW30924	5′–CTCTCTTTTGACCTATTTGGCTT–3′
LPW30925	5′–CAATCTTCAGGGGTTCCGC–3′
LPW33199	5′–TTCGCTAGTTCTCTACCGACA–3′
LPW33200	5′–AGCAGCTGCAACATGAATGAA–3′
LPW33264	5′–TTGCGCTTGCTCACCATTAGA–3′
Oligo dT-anchor primer	5′–GACCACGCGTATCGATGTCGACTTTTTTTTTTTTTTTTV–3′
